# Identification of Angiogenic Cargoes in Human Fibroblasts-Derived Extracellular Vesicles and Induction of Wound Healing

**DOI:** 10.3390/ph15060702

**Published:** 2022-06-02

**Authors:** Prakash Gangadaran, Eun Jung Oh, Ramya Lakshmi Rajendran, Hyun Mi Kim, Ji Min Oh, Suin Kwak, Chae Moon Hong, Kang Young Choi, Ho Yun Chung, Byeong-Cheol Ahn

**Affiliations:** 1 BK21 FOUR KNU Convergence Educational Program of Biomedical Sciences for Creative Future Talents, Department of Biomedical Science, School of Medicine, Kyungpook National University, Daegu 41944, Korea; prakashg@knu.ac.kr (P.G.); suin8349@naver.com (S.K.); 2 Department of Nuclear Medicine, School of Medicine, Kyungpook National University, Daegu 41944, Korea; ramyag@knu.ac.kr (R.L.R.); ojm0366@knu.ac.kr (J.M.O.); cmhong@knu.ac.kr (C.M.H.); 3 Department of Plastic and Reconstructive Surgery, CMRI, School of Medicine, Kyungpook National University, Kyungpook National University Hospital, Daegu 41944, Korea; fullrest74@knu.ac.kr (E.J.O.); sarang7939@naver.com (H.M.K.); kychoi@knu.ac.kr (K.Y.C.)

**Keywords:** fibroblasts, extracellular vesicles, miRNAs, wound healing, angiogenesis

## Abstract

A complete redevelopment of the skin remains a challenge in the management of acute and chronic wounds. Recently, the application of extracellular vesicles (EVs) for soft tissue wound healing has received much attention. As fibroblasts are fundamental cells for soft tissues and skin, we investigate the proangiogenic factors in human normal fibroblast-derived EVs (hNF-EVs) and their effects on wound healing. Normal fibroblasts were isolated from human skin tissues and characterized by immunofluorescence (IF) and Western blotting (WB). hNF-EVs were isolated by ultracentrifugation and characterized using transmission electron microscopy and WB. The proangiogenic cargos in hNF-EVs were identified by a TaqMan assay and a protein array. Other in vitro assays, including internalization assays, cell counting kit-8 analysis, scratch wound assays, WBs, and tube formation assays were conducted to assess the effects of hNF-EVs on fibroblasts and endothelial cells. A novel scaffold-free noninvasive delivery of hNF-EVs with or without fibrin glue was applied onto full-thickness skin wounds in mice. The wound healing therapeutical effect of hNF-EVs was assessed by calculating the rate of wound closure and through histological analysis. Isolated hNF was confirmed by verifying the expression of the fibroblast markers vimentin, αSMA, Hsp70, and S100A4. Isolated hNF-EVs showed intact EVs with round morphology, enriched in CD81 and CD63, and devoid of the cell markers GM130, Calnexin, and Cytochrome C. Our TaqMan assay showed that hNF-EVs were enriched in miR130a and miR210, and protein arrays showed enriched levels of the proangiogenic proteins’ vascular endothelial growth factor (VEGF)-D and CXCL8. Next, we found that the internalization of hNF-EVs into hNF increased the proliferation and migration of hNF, in addition to increasing the expression of bFGF, MMP2, and αSMA. The internalization of hNF-EVs into the endothelial cells increased their proliferation and tube formation. A scaffold-free noninvasive delivery of hNF-EVs with or without fibrin glue accelerated the wound healing rate in full-thickness skin wounds in mice, and the treatments increased the cellular density, deposition, and maturation of collagens in the wounds. Moreover, the scaffold-free noninvasive delivery of hNF-EVs with or without fibrin glue increased the VEGF and CD31 expression in the wounds, indicating that hNF-EVs have an angiogenic ability to achieve complete skin regeneration. These findings open up for new treatment strategies to be developed for wound healing. Further, we offer a new approach to the efficient, scaffold-free noninvasive delivery of hNF-EVs to wounds.

## 1. Introduction

Skin and soft tissue injuries are observed frequently in clinical settings, resulting from accidental traumas, diabetes, chronic wounds, surgery, and burn injuries [1,2,3,4,5]. Wound healing in skin and soft tissue entails an intricate biological process that requires a correct migration and proliferation of cells, followed by collagen synthesis, then collagen deposition, finally angiogenesis, and remodeling of the wound [6,7,8]. Normal wound healing can be disturbed in some pathological conditions, leading to delays in wound healing, chronically non-healing wounds such as diabetic ulcers, scars including keloid scars, and superimposed infections [8,9]. Thus, quickening wound healing time is an urgent clinical need for the prevention of chronic wounds, scar formation, and infections.

While a variety of therapeutic approaches have been developed for the advancement of wound healing, such as skin grafting, growth factor treatments, and laser therapy, all of these approaches have some drawbacks/limitation including skin necrosis and scarring, as well as the rapid degradation of topically administrated factors [10,11,12,13]. In the last few years, cell-based therapies have emerged as a novel strategy for improving wound healing. Numerous studies with promising results have shown that stem cell therapies are potentially effective at wound healing [14,15,16]. The effects of most therapeutic cells are believed to take place predominantly through paracrine factors in secreted extracellular vehicles (EVs) [17,18,19,20].

EVs are membrane-bound nano vesicles released from almost all cells in the extracellular space and culture media, and they are found in various biological fluids, such as blood, milk, urine, CNS fluids, and saliva [21]. EVs consist of exosomes (30 to 200 nm) and microvesicles (30 to 1000 nm) which are secreted by multivesicular bodies through inward budding in the cells and produced by a direct outward budding of the cell membrane, respectively. EVs play significant roles in communication pathways between distant cells that lack a direct cellular contact. EVs carry and deliver their cargos, including proteins, lipids, DNA, mRNA, miRNA, and snRNA, to recipient or target cells. This extraordinary capability of EVs of carrying bioactive cargos can be used for various medical applications [21,22].

Mesenchymal stem-cell-EVs (MSC-EVs) exhibit stem-cell-like pro-regenerative properties and have been studied in the context of regenerative therapies, such as wound healing [23,24]. However, EVs obtained from MSCs have limitations that hinder their translation into clinical settings, including donor selection, tissue source, and cellular heterogeneity [15]. To overcome these obstacles, we propose an alternative cellular method for the isolation of EVs from fibroblasts for use in wound-healing applications. Fibroblasts are easily isolated from skin, and are therefore more directly associated with skin and wound healing than MSCs [7,8].

Most studies in animal wound models that have reported using a local injection of EVs administered injections around the wound area at fewer than six sites [25,26,27,28]. This can affect the EV function in the actual wound, as it has been reported that EVs have a rapid clearance rate [29]. Most previous reports used EVs derived from either cell lines or from a mouse, and only few studies have isolated EVs from primary human cells to investigate their wound healing potential [6,25,30,31,32]. Here, we want to overcome the obstacles surrounding wound-healing therapies described in previous studies. First, we propose an alternative to MSCs and isolate normal fibroblasts from human dermal skin (hNF). Next, we explore the pro-wound healing and proangiogenic properties of hNF-derived EVs (hNF-EVs) in vitro. Finally, we investigate the therapeutic effects of a scaffold-free noninvasive delivery of hNF-EVs into a full-thickness skin wound mouse model. We believe that the current study is the first to study the effectiveness of human normal fibroblast-derived EVs in soft tissue wound healing.

## 2. Results

### 2.1. Successful Isolation and Characterization of Normal Fibroblasts from Human Skin

**Figure 1:**
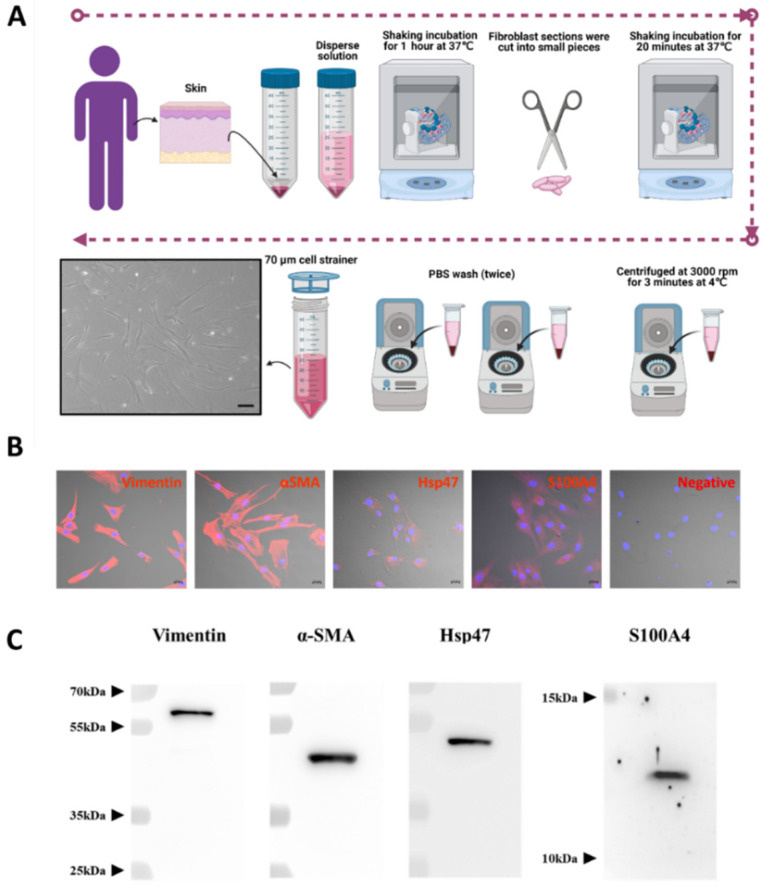
Successful isolation and characterization of normal fibroblasts from human skin. (**A**) Schematic diagram of the process used for isolating human NF (hNF) cells, created with BioRender.com (accessed on 11 August 2021) and phase-contrast imaging of hNF cells (lower left panel; scale bar: 20 µm). (**B**) Confocal microscopy of hNF cells probed with antibodies against fibroblast markers (vimentin, α-SMA, Hsp47, S100A4) and a secondary Alexa Fluor™ 555 antibody; for the negative control, no primary antibody was used (scale bar: 20 µm). (**C**) WB analysis of hNF cells with the indicated antibodies against fibroblast markers (vimentin, α-SMA, Hsp47, S100A4).

Human normal fibroblast cells (hNF cells) were isolated from human skin removed during surgery (Figure 1A). The morphology of the isolated hNF cells was observed under a light microscope and displayed an elongated shape, typical of a fibroblast-like morphology (Figure 1A, lower left panel). A fibroblast marker panel vimentin, alpha smooth muscle actin (α-SMA), heat shock protein 47 (Hsp47), fibroblast-specific protein 1 or S100 Calcium Binding Protein A4 (S100A4) was used to confirm that the isolated cells were indeed fibroblasts. Immunofluorescence assays showed that all fibroblast markers were present in isolated hNF cells, and that vimentin and α-SMA were highly expressed (Figure 1B and Supplementary Figure S1). The results from WB further confirmed that the fibroblast markers were present in isolated hNF cells (Figure 1C). Together, these data confirm that normal fibroblasts were successfully isolated and can be used for further characterization.

### 2.2. Isolation and Characterization of Human Normal Fibroblast-Derived EVs (hNF-EVs)

**Figure 2:**
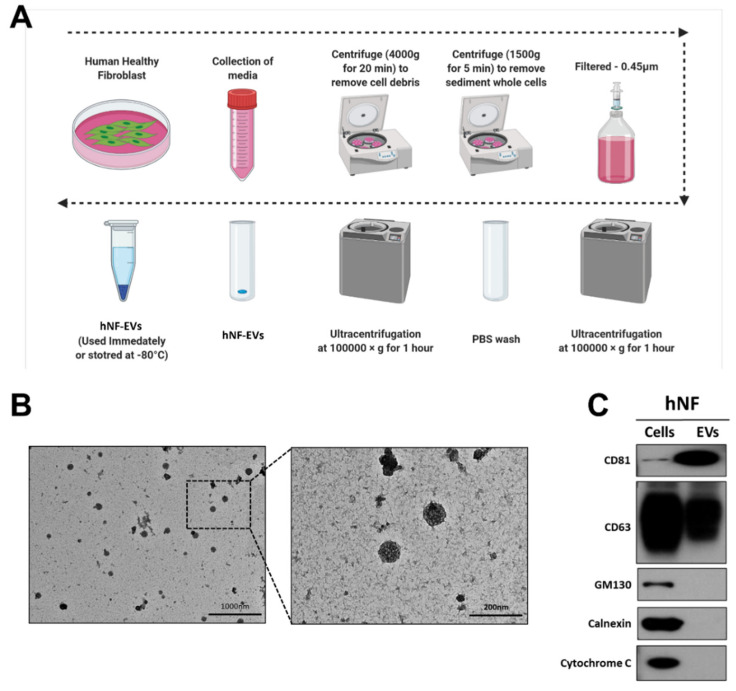
Isolation and characterization of human normal fibroblast derived extracellular vesicles (hNF-EVs). (**A**) Schematic diagram of the isolation protocol of EVs from hNF cells, created with BioRender.com (accessed on 11 August 2021). (**B**) Transmission electron microscopy of hNF-EVs; scale bar: 1000 nm and 200 nm. (**C**) Western blot (WB) analysis of hNF cells and hNF-EVs, probed with CD81, CD63, GM130, calnexin, and cytochrome C antibodies.

Fresh media isolated from a cell culture of hNFs were centrifuged, filtered, and ultracentrifuged to isolate the hNF-EVs (Figure 2A). Following the isolation of the hNF-EVs, the cell morphology was analyzed using TEM. The morphology of isolated hNF-EVs exhibits a round shape, typical of the morphology of EVs. Almost all hNF-EVs remained intact (Figure 2B). The hNF-EVs were analyzed for positive and negative biomarkers of EVs, and Western blots showed that hNF-EVs were enriched in CD81 and positive for CD63, both of which are tetraspanin protein biomarkers of EVs. Furthermore, hNF-EVs were negative for GM130 (a Golgi apparatus protein), Calnexin (an endoplasmic reticulum protein), and Cytochrome C (a mitochondrial protein), and hNF cells were positive for all three of these cell markers (Figure 2C).

### 2.3. Detection of Proangiogenic miRNAs and Proteins in HNF-EVs

**Figure 3:**
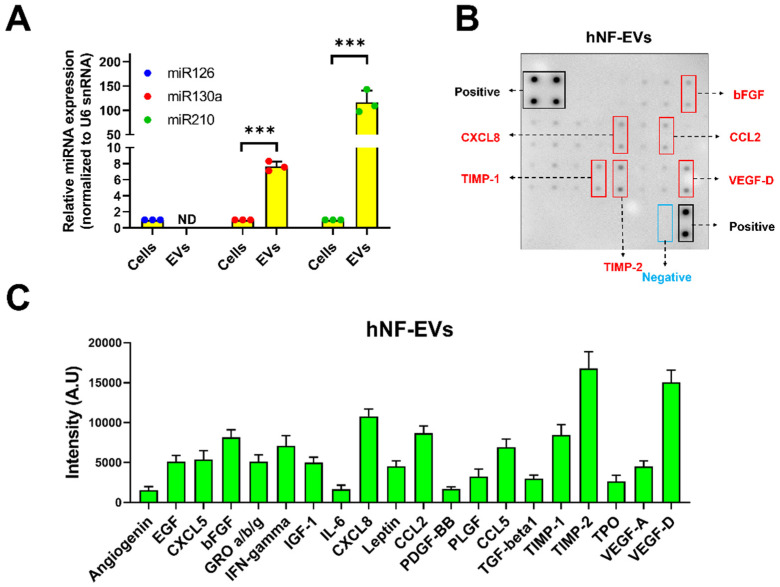
Identification of proangiogenic miRNAs and proteins in hNF-EVs. (**A**) Real-time PCR analysis for miR-126, miR-130a, and miR-210 expression in hNF cells or hNF-EVs (*n* = 3), normalized with U6 snRNA. Mean ± SD are derived from the minimum of three values from individual experiments. (**B**) A representative array blot incubated with hNF-EVs lysate (200 µg/blot). (**C**) Bar graph of the quantitative results (intensity) of the spots of each protein (*n* = 4). *** *p* < 0.001. A student’s *t*-test was used for comparison.

The presence of well-known proangiogenic miR-126, miR-130a, and miR-210 was investigated in the hNF cells and hNF-EVs. Our results demonstrated that all three miRNAs were present in the hNF cells, and two miRNAs (miR-130a and miR-210) were present in hNF-EVs. miR-126 was not detected in the hNF-EVs. The level of miR-130a was approximately 7.7-fold higher in the hNF-EVs than in their parental cells, while miR-210 was approximately 116.7-fold higher in the hNF-EVs than in parental cells (Figure 3A). Twenty angiogenic proteins in the hNF-EVs were analyzed by an angiogenic array (Figure 3B). The results demonstrated that tissue inhibitors of metalloproteinases 2 (TIMP-2) and vascular endothelial growth factor-D (VEGF-D) were present in the hNF-EVs at a higher concentration than the other proteins, followed by C-X-C Motif Chemokine Ligand 8 (CXCL8), TIMP-1, C–C motif chemokine ligand 2 (CCL2), and basic fibroblast growth factors (bFGF) (Figure 3B,C). The remaining proteins were also present but at moderate, low, or negligible levels (Figure 3B,C).

### 2.4. HNF-EVs Modulates HNF Functions In Vitro

**Figure 4:**
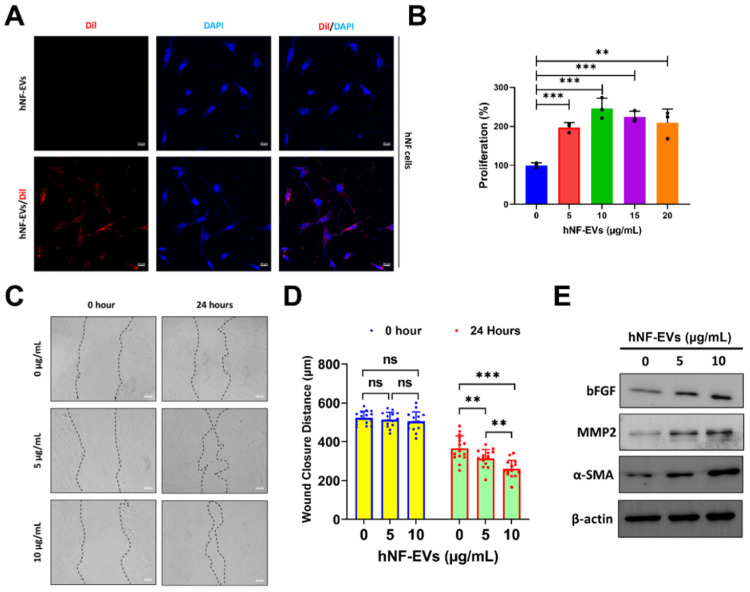
The treatment of hNF-EVs modulates hNF functions in vitro. (**A**) Fluorescent microscopy images of hNF cells treated with 10 µg/mL of hNF-EVs or hNF-EVs/DiI (scale bar: 20 µm). (**B**) The cellular proliferation of hNF cells treated with hNF-EVs (0, 5, 10, 15, and 20 µg/mL) for 24 h, quantified using a CCK8 assay kit (*n* = 3). (**C**) Representative phase-contrast images of hNF cells at 0 and 24 h after treatment with hNF-EVs (5 and 10 µg/mL), scale bar: 100 µm. (**D**) Distance measurements of wounds (C) at 0 and 24 h (*n* = 15). (**E**) WB analysis of hNF cells after 24 h of hNF-EVs treatments (5 and 10 µg/mL) probed with bFGF, MMP2, and α-SMA antibodies; β-actin was used as a loading control. ** *p* < 0.01; *** *p* < 0.001 and ns: not significant. A student’s *t*-test was used for comparison.

The interaction and internalization of EVs to recipient cells are required for the activation of receptors on cell membrane surfaces or for the delivery of cargoes into cells. Therefore, we examined the internalization of hNF-EVs into hNF cells. Fluorescence microscopy showed that hNF-EVs were well internalized into hNF cells (Figure 4A). The treatment of hNF-EVs (0, 5, 10,15 and 20 µg/mL) on hNF cells for 24 h showed that hNF-EVs were capable of significantly enhancing the proliferation of hNF cells in vitro (Figure 4B). The results from a wound-healing migration assay showed that 24 h after the hNF-EV (5 and 10 µg/mL) treatment, hNF cells significantly increased their migration at 5 µg/mL (*p* < 0.01) and 10 µg/mL (*p* < 0.001) of treatment. Furthermore, the hNF-EV (10 µg/mL) treatment significantly (*p* < 0.01) increased the migration of hNF cells compared to the treatment with 5 µg/mL (Figure 4C,D). Western blot results showed that the hNF-EV (5 and 10 µg/mL) treatment to hNF cells increased the protein levels of bFGF, MMP2, and α-SMA in a dose-dependent manner (Figure 4E).

### 2.5. Treatment of HNF-EVs Promotes In Vitro Angiogenesis

**Figure 5:**
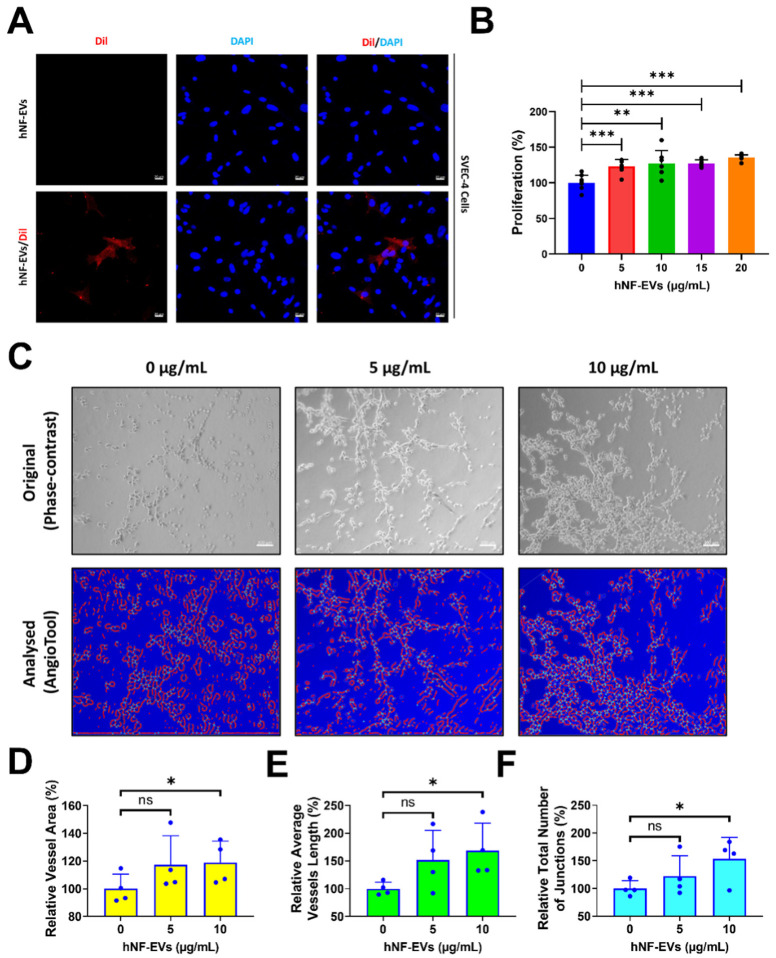
hNF-EV treatment promotes angiogenesis in vitro. (**A**) Fluorescent microscopy imaging of SVEC-4 cells treated with 10 µg/mL of hNF-EVs or hNF-EVs/DiI (scale bar: 20 µm). (**B**) The cellular proliferation of SVEC-4 cells treated with increasing concentrations of hNF-EVs (0, 5, 10, 15, and 20 µg/mL) for 24 h, quantified using a CCK8 assay kit (*n* = 6). (**C**) Original phase-contrast and analyzed images showing endothelial cells cultured on Matrigel-coated plates in media with 0, 5, or 10 μg/mL hNF-EVs (scale bar = 100 µm); (**D**–**F**) Bar graph of the relative vessel lengths, relative average vessel lengths, and relative total number of junctions measured in the field of view (*n* = 4) using AngioTool64 software, (National Cancer Institute, Radiation Oncology Branch, Angiogenesis Core Facility, MD, USA) Version 0.6a (02.18.14). * *p* < 0.05; ** *p* < 0.01; *** *p* < 0.001 and ns: not significant. A student’s *t*-test was used for comparison.

Fluorescent microscopy imaging showed that hNF-EVs are well-internalized into SVEC-4 (endothelial cells) (Figure 5A). The treatment of hNF-EVs (0, 5, 10,15 and 20 µg/mL) to SVEC-4 cells for 24 h showed that hNF-EVs are capable of significantly (*p* < 0.001; 0 vs. 5, 10, 15, and 20 µg/mL) enhancing the cellular proliferation of SVEC-4 cells in vitro (Figure 5B). The in vitro tube formation effect of hNF-EVs (5 and 10 µg/mL) was tested on SVEC-4 cells under Matrigel conditions. The assay results showed that hNF-EVs enhanced the tube formation of SVEC-4 cells (Figure 5C). The AngioTool analysis further revealed that the hNF-EV (5 µg/mL) treatment substantially increased the relative vessel area, whereas a higher hNF-EV (10 µg/mL) treatment significantly (*p* < 0.05) increased the vessel area compared to the control (Figure 4D). The hNF-EV (5 µg/mL) treatment substantially increased the average vessel length, whereas higher hNF-EVs (10 µg/mL) significantly (*p* < 0.05) increased the average vessel length compared to the control (Figure 5E). The hNF-EV (5 µg/mL) treatment substantially increased the total number of junctions, whereas the hNF-EV (10 µg/mL) treatment significantly (*p* < 0.05) increased the total number of junctions compare to the control (Figure 5F).

### 2.6. HNF-EVs Treatment Promotes Cutaneous Wound Healing in Mice

**Figure 6:**
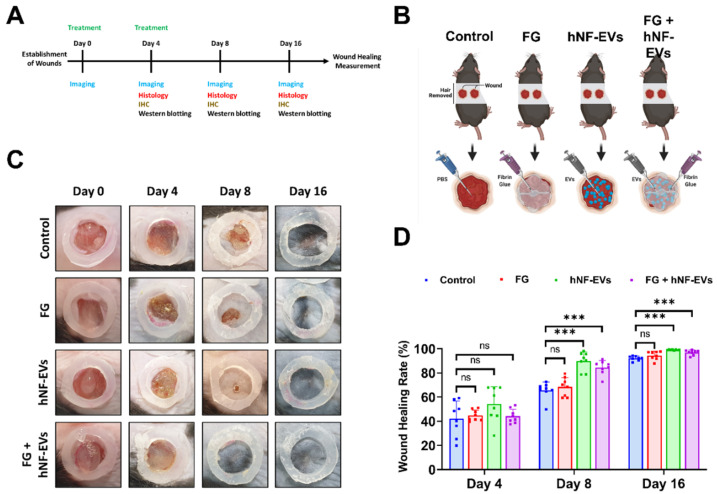
hNF-EVs treatment of accelerated wound-healing in C57BL/6 mice. (**A**) Schematic of in vivo experiments. (**B**) A schematic representation of in vivo treatments (Control: PBS; Fibrin Glue: FG; hNF-EVs; FG + hNF-EVs), created with BioRender.com accessed on 13 April 2021. (**C**) Representative images display the wound healing process of C57BL/6 mice treated with control: PBS; Fibrin Glue: FG; hNF-EVs; FG + hNF-EVs (day 0, 4, 8, and 16). (**D**) Percentage of wound healing in mice treated with control: PBS; Fibrin Glue: FG; hNF-EVs; FG + hNF-EVs (mice = 4 or wounds: *n* = 8). *** *p* < 0.001 and ns: not significant. A student’s *t*-test was used.

Next, we investigated the effects of hNF-EVs, FG, and a combination treatment on skin wound healing by creating a full-thickness cutaneous wound on the dorsal skin of mice. We analyzed photographs of wounds and performed WB and histological analysis for further evaluation (Figure 6A). The treatment of controls (PBS), FG, hNF-EVs and FG + hNF-EVs was applied as illustrated (Figure 6B). On day 4, wound healing was not significantly (*p*  >  0.05) accelerated in FG, hNF-EVs, or the combination FG + hNF-EVs-treated mice compared to the control group. On day 8, wound healing was slightly accelerated in FG-treated mice compared to the controls and significantly (*p*  <  0.001) accelerated in hNF-EVs and FG + hNF-EVs-treated mice compared to the controls. Similarly, on day 16, wound healing was slightly accelerated in FG-treated mice and significantly (*p*  <  0.001) accelerated in hNF-EVs and FG + hNF-EVs-treated mice compared to the control mice. (Figure 6C,D; Supplementary Figure S2A).

### 2.7. HNF-EV Treatment Increases Cutaneous Cellular Density and Accelerates Collagen Deposition and Maturity in Mice Wounds

**Figure 7:**
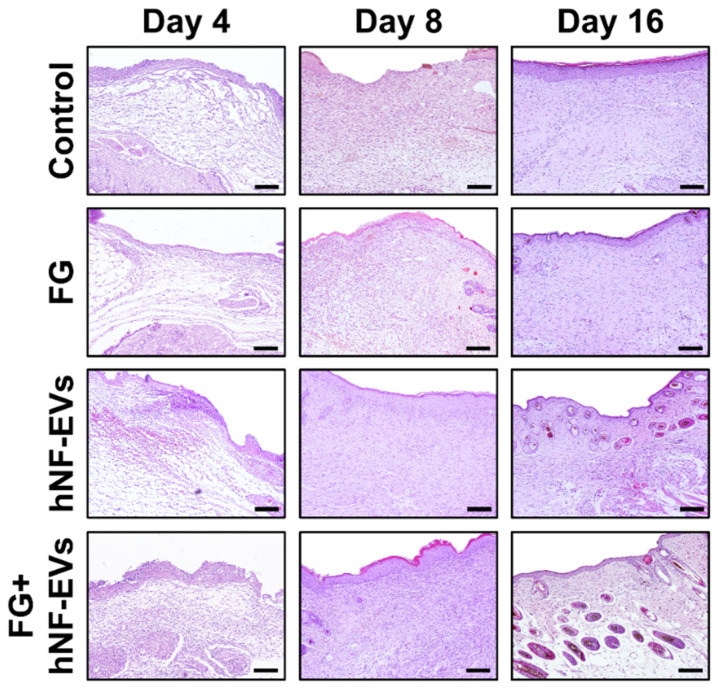
hNF-EVs promoted skin density. H&E staining of wound sections in control: FG, hNF-EV, and FG + hNF-EV groups (day 4, 8, and 16; scale bar = 100 μm).

**Figure 8:**
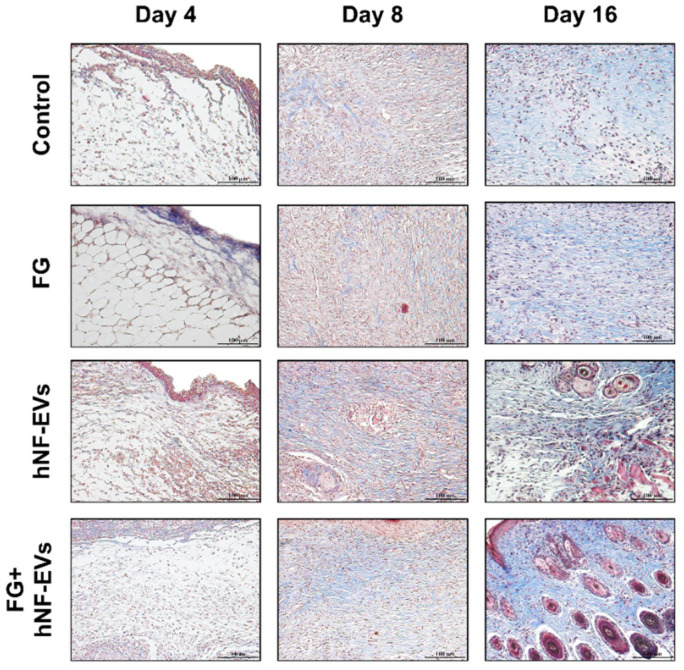
hNF-EVs accelerate collagen deposition and maturity in the wound. Masson’s trichome staining of wound sections in controls: FG, hNF-EV, and FG + hNF-EV groups (day 4, 8, and 16), (scale bar = 100 μm).

The H&E staining of excised tissue sections showed tightly filled cells in the wound area in both the hNF-EVs and FG + hNF-EVs treatment groups compared to the PBS- and FG-treated groups at days 4 and 8. White space was observed (unreconstructed area) more in the dermis of controls and FG-treated wounds than in hNF-EVs and FG + hNF-EVs in both staining techniques (H&E and Masson’s trichrome) (Figure 7 and Figure 8) at day 4. By day 8, the H&E staining of wounds in the control and FG treatment groups showed a higher number of infiltrating inflammatory cells compared to the hNF-EV- and FG + hNF-EVs-treated wounds. Finally, on day 16, H&E staining showed that the hNF-EV- and FG + hNF-EVs-treated wounds had more regenerated hair follicles than those of controls and FG-treated mice (Figure 7). The hNF-EVs and FG + hNF-EVs had an excellent epidermal maturation, while the control and FG-treated wounds showed only a partial epidermal maturation (Figure 7). In addition to these observations, skin appendages could be seen clearly on day 16 of hNF-EV- and FG + hNF-EVs-treated wounds, while they could not be observed for control and FG-treated wounds. (Supplementary Figure S2B). Masson’s trichrome staining showed a significantly higher percentage of collagen fibers in the wounds treated with hNF-EVs (~17%) and FG + hNF-EVs compared to control and FG-treated wounds (Figure 8).

### 2.8. HNF-EV Treatment Enhances Angiogenesis in the Wound Sites of Mice

**Figure 9:**
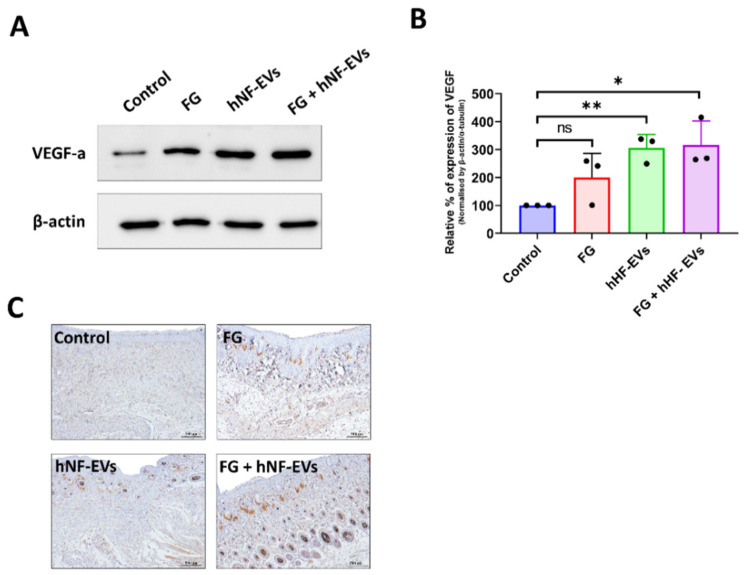
The promotion of angiogenesis by the hNF-EV treatment in wounds of mice. (**A**) Western blot analysis of VEGF and CD31 levels in wounds at day 16 treated with controls, FG, hNF-EVs, and FG + hNF-EVs. β-actin was used as a loading control. (**B**) Quantification of the band intensity of VEGF in the wound skin of mice treated with control, FG, hNF-EVs, and FG + hNF-EVs at day 16 (mice: *n* = 2 or wound: *n* = 3) using the GelQuantNet software; the mean and the SD of experiments are plotted. (**C**) CD31 IHC staining in wound sections treated with control, FG, hNF-EVs and FG + hNF-EVs at day 16 (scale bar = 500 μm). * *p* < 0.05; ** *p* < 0.01 and ns: not significant. A student’s *t*-test was used.

Next, we asked whether the treatment of hNF-EVs could enhance angiogenesis in the wounds. First, the expression of VEGF in the wound was examined by WB at day 16. The results from the immunoblots showed that VEGF expression was increased in all three treated wounds compared to the control wounds (Figure 9A), and the quantification demonstrated that the significantly increased levels of expression of VEGF-a were about 316% in the FG + hNF-EVs (*p* < 0.05), followed by about 305% in the hNF-EVs (*p* < 0.01), and substantially increased by about 200% in the FG-treated wounds compared to the controls (Figure 9B). Immunohistochemistry staining for CD31 was performed to detect blood vessels in the wounds. Blood vessels were hardly evident in the control or FG wounds, whereas substantially more blood vessels were observed in the hNF-EVs and FG + hNF-EVs treated wounds (Figure 9C).

## 3. Discussion

Recent research on the utility of EVs in regenerative medicine has gained great attention and is being studied by regenerative-medicine-related researchers. Currently, many researchers believe that EVs could be used as a potential therapeutic strategy for wound healing, as an alternative to stem-cell-based therapies. Most of the previous studies reporting on wound healing and angiogenesis used MSCs as a source of EVs; EVs were then derived from mouse origins or cell lines [6,17,23,25,33]. This became a major hurdle to the translation of EV-based therapies for wound healing into the clinic. Here, we used primary human fibroblasts derived from skin as a novel, alternative source for EVs. The isolated normal human fibroblasts showed a typical fibroblast morphology, and they were strongly positive for fibroblast markers including vimentin, αSMA, hsp47, and S100A4, in agreement with previous studies [34,35]. The hNF-EVs exhibited a typical EV round shape morphology, indicating they were intact. The isolated hNF-EVs showed an enrichment of the EV markers CD81 and CD63. Moreover, hNF-EVs were uncontaminated by cells/cellular organelles, confirmed by the absence of the Golgi marker (GM130), endoplasmic reticulum marker (Calnexin), and mitochondrial marker (Cytochrome C). Together, these results confirm that hNF-EVs were intact, enriched, and uncontaminated with cellular debris, consistent with previous reports [6,33,36].

A large number of studies reported that EVs consist of large amounts of miRNAs, and that these miRNAs can be transferred to recipient cells and lead to biological alterations in recipient cells [37]. Angiogenesis is a crucial factor in the capability of a tissue to repair itself through the development of a new vasculature to supply nutrients and oxygen to the wound [38]. We have showed as much in our previous study with a cell line of mouse fibroblast-derived EVs on accelerated wound healing effects, but we failed to show which component of EVs (such as miRNAs and/or proteins) may be fully or partially responsible for the effects. Therefore, in the current study, we investigated whether our hNF-EVs possess any well-known proangiogenic miRNAs, such as miR126, miR130a, and miR210). Our results demonstrated that miR130a and miR210 were present in hNF-EVs, while miR126 was not detectable in hNF-EVs. miR130a and miR210 were not only present in hNF-EVs, but also more enriched in hNF-EVs than in hNF cells. The miR130a is known to target GAX and HOXA5 genes (antiangiogenic homeobox genes) in endothelial cells to activate angiogenesis. In addition, exosome-derived miR130a targets C-MYB to induce angiogenesis in endothelial cells, and our recent study with macrophage-derived EVs’ enriched with mi130a promoted angiogenesis both in vitro and in vivo [18,39,40]. In hNF-EVs, the miR210 were enriched more than 100-fold relative to miR210 in hNF cells. Our previous reports showed that MSC-EVs enriched with miR210 promoted angiogenesis in vitro and revascularized ischemic hindlimbs in a mouse model [20]. In addition, another report suggests that miR210 may influence the VEGF signaling pathway and promote angiogenesis. miR210 has also been shown to promote angiogenesis in acute myocardial infarction [41,42]. Finally, a recent study showed that local miR-210 administration accelerated wound healing in diabetic mice [43]. As mentioned above, angiogenesis is crucial for tissue repair, so hNF-EVs could be a potential therapeutic candidate for regenerative therapies, including in wound healing.

EVs carry not only nucleic acids, but also proteins both on the EV membrane and inside EVs. Thus, twenty angiogenic proteins were investigated in hNF-EVs derived from two separate humans. Our angiogenic protein array found that most angiogenic proteins are present in hNF-EVs at different levels, in agreement with our previous report [36]. Among the known angiogenic proteins, TIMP-1 and VEGF-D were present at the highest level. TIMP-1 is known to regulate angiogenesis and is secreted via EVs [36,44,45]. VEGF-D is well known for its role in activating VEGFR2/3 and serves as the greatest activator of angiogenesis and lymphangiogenesis [36,46,47]. After TIMP-1 and VEGF-D, CXCL8, which directly enhances the survival and proliferation of endothelial cell and increases the production of matrix metalloproteinases, was the next most abundant protein present in hNF-EVs that can induce angiogenesis [48,49]. Lastly, bFGF, which induces the proangiogenic properties of fibrocytes and accelerates blood vessel formation during the wound-healing process, was also detected in hNF-EVs [50].

Fibroblasts are the most important cells in soft tissue wound healing, and their cellular proliferation and migration to the wound are essential for contraction of wound, synthesis of collagen, and finally, remodeling of tissue [51]. In the current study, the treatment of hNF-EVs to hNF cells increased the proliferation and migration of hNF cells to a large degree, in agreement with our previous study showing that mouse-derived fibroblast EVs increased the proliferation and migration of mouse fibroblast cells. In addition, when compared to a previous study of mouse-derived fibroblast EVs, the current hHF-EVs treatment showed a 2- to 2.5-fold increase in the proliferation of fibroblasts with a lower concentration of EVs, whereas mouse-derived fibroblast EVs showed a 0.5-fold increase in proliferation. Similarly, the migration was also faster in the hHF-EV treatment than in mouse-derived fibroblast EVs [6]. In addition, a Western blot analysis showed that the hNF-EV treatment increased bFGF, MMP2, and αSMA in recipient hNF cells. The bFGF increase in fibroblasts is known to be associated with enhancing wound healing through the induction of angiogenesis [50]. The increase in MMP2 and its subsequent secretion induces the migration of keratinocytes at the wound matrix, and previous reports have demonstrated that MMP2 can induce angiogenesis through the stimulation of tube formation in endothelial cells [52,53]. In healing infarcts, scar contraction is associated with the accumulation of αSMA-expressing myofibroblasts [54,55], and our result showed that the hNF-EV treatment increased the αSMA levels in hNF cells. Altogether, these findings indicate that hNF-EVs are capable of promoting fibrogenesis.

In the wound area, the proliferation of skin cells at the margin of the wound is required for generating new tissues and is assisted by the vascularization in the newly generated tissue. The angiogenesis process starts by development of new blood vessels from the pre-existing blood vessels [36,56,57]. Our results showed that the hNF-EV treatment of endothelial cells leads to cellular proliferation in vitro. In addition, the hNF-EV treatment also increased the vessel area, the average vessel length, and the number of junctions in the vessels in vitro, and these results showed no differences in the pro-angiogenesis effects of hHF-EVs in endothelial cells compared to our previous study with mouse-derived fibroblast EV treatments [6]. Many earlier studies reported that miR130a and miR210 are prolific mediators of angiogenesis, particularly for endothelial cell proliferation and blood vessel-like formation [39,40,41,42,43]. Therefore, hNF-EVs consisting of these miRNAs may regulate angiogenesis positively. In addition, hNF-EVs contain VEGF-D, CXCL8, and bFGF, which are factors well known to promote angiogenesis by regulating endothelial cells [46,47,48,50,56]. Other miRNAs or proteins in hNF-EVs cannot be excluded as contributors to enhanced angiogenic activity.

We investigated the therapeutic effects of hNF-EVs on an in vivo wound mouse model. Almost all previous studies used a local injection as the delivery method in wound-healing models [25,26,27,28]. This method may result in a minimal accumulation within the desired wound sites, and a rapid clearance rate may result in a low amount of EVs at the injected site [29]. Here, we proposed a scaffold-free noninvasive delivery method of hNF-EVs to wounds, and compared the efficiency of the noninvasive method with the hNF-EVs + FG method to retain hNF-EVs in the target site. FG is made from a mix of two components, fibrinogen and thrombin, and FG is often used in real world clinics in order to stop bleeding, as it has gelation properties and degrades naturally [6].

We established a novel, scaffold-free noninvasive delivery method by applying hNF-EVs to the wound area of mice by pipetting small volumes. We made hNF-EVs in PBS to give them a gel-like consistency. We also combined FG and hNF-EVs, which we believe may further help retain hNF-EVs at the wound site. We used PBS and FG alone as controls. We found that a scaffold-free noninvasive delivery of hNF-EVs as treatment accelerated healing of a wound in an in vivo model (mouse), which is about 90% healing of the wound in 8 days, with an FG + hNF-EVs treatment at 85%, FG at 68%, and the control at 66% after 8 days. On day 18, the hNF-EV and FG + hNF-EV groups showed complete healing compared to the FG and control groups. These results suggest that both noninvasive deliveries, with FG and without FG, have a significant therapeutic potential in wound healing. To our surprise, the hNF-EVs showed a much earlier acceleration of wound healing than the FG + hNF-EV groups, perhaps because, immediately after the scaffold-free noninvasive delivery of hNF-EVs, the wound area took up hNF-EVs more effectively, as the surface area of contact between hNF-EVs and the wounds was larger than that of the injection of EVs at a few sites [25,26,27,28]. In the case of the FG + hNF-EV group, hNF-EVs may be released more substantially than the hNF-EV group. In our recent study, we used the delivery of EVs with FG [6] only, but now we show that a scaffold-free noninvasive delivery of hNF-EVs without gels (i.e., FG) can be effective in wound healing. We believe that the current study is the first study to demonstrate effectiveness of the scaffold-free noninvasive delivery of hNF-EVs in wound healing.

H&E and Masson’s trichrome staining sections showed the formation of well-organized epidermis, dermis, and skin appendages in the scaffold-free noninvasive treatment of hNF-EV and FG + hNF-EV groups compared to controls, further confirming the complete recovery of the skin and a scarless wound healing. Appropriate collagen deposition and remodeling could improve the tissue stretching potency and result in better wound repair and regeneration [58,59,60]. We observed that wounds treated with hNF-EVs and FG + hNF-EVs showed appropriate and relatively well-organized collagen fibers at day 8. The excessing collagen deposition may lead to scar formation in the wound site. Even though collagen deposition was accelerated by hNF-EVs, the effects of EVs in the wound site are transient. As such, collagen deposition may not lead to excess collagen deposition in the wound to induce scars. A few studies with exosomes (small EVs) or EVs from stem cells induced an appropriate collagen deposition and reduced the scar formation [61,62,63]. Further studies are required to study the excessing collagen deposition by hHF-EVs. Our Western blot results on the skin of treated wounds showed that the FG + hNF-EV treatment increased the levels of VEGF, followed by the VEGF levels in hNF-EVs and FG, compared to the control. We also observed that hNF-EVs contained enriched proangiogenic factors including miR210, which is consistent with a previous study showing that the upregulation of miR210 regulates the activation of the VEGF signaling pathway under ischemia or perfusion injuries in vivo [41]. An increase in VEGF eventually leads to the formation of blood vessels that deliver nutrients and oxygen to new tissues. The results of IHC validated the blood vessel formation and enhanced the following treatments. Overall, the in vivo accelerated wound healing effects of hHF-EVs are comparable to our previous study with mouse-derived fibroblast EVs treatments. The current study results are not superior to those of mouse-derived fibroblast EV treatments in vivo [6]. This may be due to species-to-species changes, as the in vivo model is from mice and the hHF-EVs are of human origin. Further, clinical trials with human skin are required to verify the effects of human fibroblast-derived EVs in wound healing.

The limitations of using a fibroblast as a source for the isolation of EVs include the limited supply of cells with consistent quality, as fibroblasts exhibit anatomical heterogeneity and replicative lifespan, depending on the individual [64]. Engineering the fibroblasts with potent wound healing effector genes or differentiation fibroblasts from embryonic stem cells may be used to collect the EVs for wound healing, which will overcome the limitations of fibroblasts for EV sources.

## 4. Materials and Methods

### 4.1. Human and Animal Ethics Approval Statement

This study was approved by the Institutional Review Board (IRB) of Kyungpook National University Hospital (IRB NO: KNUH 2019-07-023-001) and performed in accordance with the principles of the Declaration of Helsinki. Informed consent was obtained from all subjects involved in the study. All procedures were reviewed and approved by Kyungpook National University’s Animal Care and Use Committee (IRB NO: KNU-2020-0076) and were performed in accordance with the Guiding Principles for the Care and Use of Laboratory Animals.

### 4.2. Isolation of Human Primary Fibroblasts and Culture

Human excess skin from full-thickness skin grafts was harvested from the groin area during surgery, and the Dispase II (Gibco, Carlsbad, CA, USA) solution was added to the skin for 3 h in order to remove the epidermis. The skin was kept in a shaking incubator for 1 h at 37 °C to remove the epidermis and fat. Fibroblast sections were cut into small pieces using scissors and kept in a shaking incubator for 20 min at 37 °C. Then, the samples were centrifuged at 3000× *g* rpm for 3 min at 4 °C, the supernatant was discarded, and the remaining cells were washed twice with PBS. The cells were separated with a 70 µm cell strainer (Corning^®^, Corning, NY, USA). They were cultured in DMEM (HyClone, Logan, UT, USA) supplemented with 10% EV-depleted fetal bovine serum (FBS; HyClone) (depleted by centrifugation at 120,000× *g* for 18 h at 4 °C) and 1% penicillin–streptomycin (Gibco, Carlsbad, CA, USA) at 37 °C in the presence of 5% CO_2_. The cells were used up to five passages in this study.

### 4.3. Endothelial Cell Culture

Mouse endothelial cells (SVEC-4) were cultured in DMEM (HyClone) supplemented with 10% EV-depleted FBS (HyClone) (depletion performed by centrifugation at 120,000× *g* for 18 h at 4 °C) and 1% penicillin–streptomycin (Gibco) at 37 °C in the presence of 5% CO_2_.

### 4.4. Western Blotting (WB)

WB was performed as described previously [65]. Briefly, whole cells or EV lysates were prepared using a radio immune-precipitation assay buffer (Thermo Fisher Scientific, Waltham, MA, USA) supplemented with a protease inhibitor cocktail (Sigma-Aldrich, St. Louis, MO, USA). Equal amounts of proteins were loaded onto 10% SDS-PAGE gels. The proteins were transferred from the gel to polyvinylidene difluoride (PVDF) membranes (Millipore, Burlington, MA, USA), and the membranes were blocked with 5% milk and probed with primary antibodies overnight at 4 °C (vimentin (Abcam, Cambridge, UK; 1:1000), α-SMA (Abcam; 1:1000), Hsp47 (Abcam; 1:1000), S100A4 (Abcam; 1:1000), CD63, (Abcam; 1:5000), CD81, (Abcam; 1:5000), cytochrome C (Abcam; 1:5000), GM130, (Abcam; 1:4000), calnexin, (Abcam; 1:5000), (Abcam; 1:5000), bFGF, (CST; 1:2500), MMP2, (Abcam; 1:2500), vascular endothelial growth factor (VEGF)-a, (Abcam; 1:2500), CD31, (Abcam; 1:2000) and β-actin, (CST; 1:10,000)). The blots were incubated with appropriate secondary antibodies (CST) conjugated with horseradish peroxidase (HRP). Antibody signals were detected using ECL+ solution (GE Healthcare, Chicago, IL, USA), and protein bands were visualized using a Fusion FX chemiluminescence analyzer system (Vilber Lourmat, Marne-la-Vallée, France) or Medical X-ray films (Afga NV, Septestraat, Mortsel, Belgium). Blot images were cropped and prepared using Microsoft PowerPoint (Microsoft, Redmond, WA, USA). The band intensities were measured using GelQuant.NET software (Biochem Lab Solutions, San Francisco, CA, USA).

### 4.5. Immunofluorescence Assay

For immunofluorescence studies, 2 × 10^4^ human fibroblast cells were seeded per well into 4-well chamber slides and grown overnight. The next day, the cells on slides were fixed in 2% paraformaldehyde. They were blocked with 3% BSA for 30 min, incubated with vimentin (Abcam; 1:200), α-SMA (Abcam; 1:200), Hsp47 (Abcam; 1:200), and S100A4 (Abcam; 1:200) overnight, and then washed three times with PBS. The cells were incubated with a secondary antibody conjugated with Alexa Fluor™ 555 (Cell Signaling Technology) for 1 h, washed again three times in PBS, and mounted in a DAPI-containing mounting medium (Vector Laboratories, Burlingame, CA, USA). The cells were imaged with a Zeiss super-resolution confocal microscope (LSM 5 Exciter, Carl Zeiss, Baden-Württemberg, Germany).

### 4.6. Isolation and Purification of EVs from Fibroblast Cells

Human fibroblast cells were cultured in an EV-depleted, FBS-containing medium (ultracentrifuged for 18 h at 120,000× *g* and 4 °C). The human fibroblast cells were grown on 100 mm plates (Thermo Fisher Scientific) and, as per standard procedure, cultured media harvested when cells were at 85–95% confluence. The cultured medium was used for the isolation of EVs. First, the culture medium was centrifuged at 1500× *g* for 5 min and then at 4000× *g* for 20 min to remove the sediments of whole cells and cell debris, respectively. The collected supernatant was filtered through a 0.45 μm filter to remove the larger vesicles. The filtered medium was collected and ultracentrifuged at 100,000× *g* for 1 h. The supernatants were discarded, and EV pellets were resuspended in PBS followed by ultracentrifugation at 100,000× *g* for 1 h. The EV pellets were used directly or stored for later use (−80 °C). All centrifugations and ultracentrifugation were performed at 4 °C. The concentration of EVs was measured using a Pierce BCA Protein Assay Kit (Thermo Fisher Scientific).

### 4.7. Transmission Electron Microscopy

Transmission electron microscopy was performed as described previously [66]. Briefly, hNF-EVs pellets were resuspended in 100 μL of 2% paraformaldehyde. Then, 5 μL of hNF-EVs was placed onto a parafilm, and a formvar-carbon coated EM grid was placed on the sample to absorb the surface of the grid. Then, the samples were covered and incubated for 20 min. The hNF-EVs were then washed by placing a 100 μL drop of PBS pipetted directly onto the parafilm, inverting the sample, and placing it over the grid with clean forceps. The grid was then incubated in a 50 µL drop of 1% glutaraldehyde for 5 min, before being washed seven times for 2 min in distilled water. The sample was examined, and images were captured using a HT 7700 transmission electron microscope (Hitachi, Hitachi-shi, Ibaraki-ken, Japan) operated at 100 kV.

### 4.8. RNA Isolation and cDNA Conversion

Cell or EV pellets were lysed using the TRIzol^®^ reagent (Sigma-Aldrich, St. Louis, MO, USA), and the total RNA was extracted according to the manufacturer’s instructions. An equal amount of RNAs from cells and EVs were used for a reverse transcription of miRNAs, performed using the High-Capacity cDNA™ Kit (Thermo Fisher Scientific) according to the manufacturer’s instructions.

### 4.9. TaqMan Assay

The expression of miRNAs in hNF cells and hNF-EVs was quantified using a TaqMan™ Universal Real-Time PCR Assay with a ABI7500 detection system (Applied Biosystems, Foster, CA, USA), according to the manufacturer’s instructions. An equal volume of cDNA was used for all samples. Using the TaqMan™ MicroRNA Assays, miR-126-5p, miR-210-3p, and miR-130a were detected. The miRNA expression was normalized to the U6 snRNA expression, and the fold change of the expression between hNF cells and hNF-EVs was calculated using 2^−ΔΔCt^.

### 4.10. Human Angiogenesis Array

Proteins extracted from hNF-EVs (200 µg/blot) of two human subjects were used for this assay. The Human Angiogenesis Array (RayBiotech, Peachtree Corners, GA, USA) was used according to the manufacturer’s instructions. The intensity was measured with the GelQuant.NET software (Version 1.8.2) (Biochem Lab Solutions).

### 4.11. EVs Labeling and Internalization Assay

The lipophilic Dil Stain (1,1′-Dioctadecyl-3,3,3′,3′-Tetramethylindocarbocyanine Perchlorate (‘DiI’; DiIC_18_(3))) (Thermo Fisher Scientific) was mixed with hNF-EVs for 20 min at 37 °C in a shaking incubator. DiI-labeled hNF-EVs (hNF-EVs/DiI) were isolated by an Exo-Quick solution (System Biosciences, Palo Alto, CA, USA) according to manufacturer’s instructions, and then the mixture of Dil and hNF-EVs was diluted with PBS and Exo-Quick solution was added to mixer and incubated in 4 °C for 1 h. Then, the sample was centrifuged at 3000× *g* for 5 min at 4 °C to isolate the EVs (hNF-Evs/DiI) as pellets and free Dil in supernatant, then the supernatant was discarded. The pellets were reconstituted with PBS and the Exo-Quick EV isolation steps were repeated again to remove remaining free DiI. Then, the pellets were reconstituted with PBS and used for internalization assay.

The hNF cells or SVEC-4 cells (2 × 10^4^) were seeded into 8-well chambers and incubated overnight in a CO_2_ incubator. Then, 10 µg/mL of unlabeled hNF-EVs and DiI-labeled hNF-EVs (hNF-EVs/DiI) were incubated with hNF cells or SVEC-4 cells for 1 h. The cells were washed with PBS and fixed with 2% paraformaldehyde for 10 min. They were washed again with PBS. Slides were mounted in an anti-fade mounting medium with DAPI (Vectashield, Burlingame, CA, USA). The internalization of hNF-EVs was imaged using a super resolution confocal laser scanning microscope, the LSM 800 with Airyscan (Carl Zeiss, Baden-Württemberg, Germany).

### 4.12. In Vitro Cellular Proliferation Assay

The hNF cells or SVEC-4 cells (1 × 10^4^) were seeded into 96-well plates in a complete medium and grown overnight in a CO_2_ incubator. The next day, the hNF-EVs (0, 5, 10, 15, and 20 µg/mL) were added to wells with a fresh complete medium and kept in a CO_2_ incubator for 24 h. A volume of 10 µL of CCK8 solution (CCK8 assay kit, Dojindo Molecular Technologies, Kyushu, Japan) was added to each well. The cells were incubated for 2 h in a CO_2_ incubator, and according to the manufacturer’s protocol, the cellular proliferation was measured by measuring the optical density (450 nm) by a spectrophotometer. 

### 4.13. In Vitro Wound Healing Migration Assay

The hNF or SVEC-4 cells (5 × 10^5^) were grown on 35-mm plates at 5% CO_2_ until they reached a 90% to 95% confluence. Using a sterile 10-μL pipette tip, a scratch wound was made. The detached cells were removed using PBS wash and replaced with a fresh serum-free medium. The scratch wound healing was captured at time 0 by an AXIO microscope (Zeiss, Baden-Württemberg, Germany). Then, the cells were treated with hNF-EVs (5 and 10 μg/mL). The scratch wound healing was captured again at 12 or 24 h with the AXIO microscope. The open wound distance was estimated from five measurements in each microscopic field of view using ZEN lite 2.3 (Carl Zeiss, Baden-Württemberg, Germany).

### 4.14. In Vitro Matrigel Tube Formation Assay

SVEC4 cells (7.5 × 10^4^/well) were seeded onto 24-well plates coated with a Matrigel Growth Factor Reduced Basement Membrane Matrix (Corning, Tewksbury, MA, USA). Immediately after cell seeding, the hNF-EVs (0, 5 and 10 µg/mL) were added to the appropriate wells. Tube formation was monitored and imaged 4 h after treatment with an AXIO microscope (Zeiss). The relative vessel area, relative average vessel length, and relative total number of junctions were counted automatically using the AngioTool64 software (National Cancer Institute, Radiation Oncology Branch, Angiogenesis Core Facility, MD, USA) [67].

### 4.15. Establishment of a Wound Healing Mice Model and Scaffold-Free Noninvasive Delivery hNF-EVs

C57BL/6 mice (6 weeks old, female) were purchased (Hana, Busan, Korea) and used for the evaluation of the wound-healing effects by fibrin glue, hNF-EVs, and a combination. First, the mice were anesthetized using 2.5% of isoflurane (Merial, Lyon, France). The hairs were removed using an electrical shaver, and after sterilization using povidone-iodine and 70% alcohol, the wound areas were marked on both sides of the mouse’s back with a distance of 1.5 mm. After making an incision with a circular biopsy punch instrument with a diameter of 8 mm (Kai Medical, Seki, Japan), the skin inside the incision circle was separated from the subcutaneous layer using Metzenbaum scissors, and circular full-thickness skin wounds with 8 mm of diameter were made on each side of the mouse’s back (2 wound/mouse). A silicone ring with an inner diameter of 8 mm and an outer diameter of 12 mm was glued to the wound to avoid any contraction (Dermabond, Ethicon, New Brunswick, NJ, USA). The mice were randomly divided into four groups: control (*n* = 10), fibrin glue (FG, *n* = 10), hNF-EVs (*n* = 10) and FG + hNF-EVs (*n* = 10). On the day of treatment, the control group received 20 μL of PBS to their wounds, the FG group received 100 μL of fibrin glue (Greenplast Q, GC Pharma, Yongin, Korea), the hNF-EV group received 100 μg hNF-EVs in 20 μL of PBS, and the FG + hNF-EV group was treated with 100 μg of hNF-EVs in 20 μL of PBS and 100 μL of fibrin glue. All wounds were covered immediately with sterile gauze and Tegaderm transparent film (3 M, London, UK), and elastic support bandages were used to cover the wounds further. In general, all groups underwent wound dressing once a day until the fourth day; postoperatively, once every 2 days from day 4 to 10; and every 3 to 4 days after the 10th day.

### 4.16. Measurement of Wound Healing and Collection of Wounded Tissues for WB

To measure the size of the wounds on days 0, 4, 8, and 16, the Visitrak grid films and the Visitrak digital wound analysis system (Smith & Nephew, London, UK) were used as described previously [68]. The percentage of wound healing was determined according to the following equations: Wound healed (%) = (W0 − Ui)/W0 × 100, wound contraction (%) = (W0 − Wi)/W0 × 100, and wound epithelialization (%) = (Wi − Ui)/W0 × 100. On days 4, 8, and 16, wound tissues were collected for WB and histology. For the Western blots, the tissues were chopped into small pieces and homogenized. Total proteins were isolated using the commercial total protein isolation kit (iNtRON Biotechnology, Seongnam-si, Korea) from homogenized tissue samples and processed for WB.

### 4.17. Histology and Measurements

The skin tissues of all groups were fixed (10% formalin buffer). The paraffin-embedded blocks were prepared, and five-micron sections were embedded on the glass slides. The tissue sections were processes to H&E or Masson’s trichrome staining, and tissue sections were imaged under an inverted light microscope (Leica Microsystems, Wetzlar, Germany).

### 4.18. Immunohistochemistry (IHC)

IHC staining of CD31 and CD68 was performed as described previously [69] using a DAKO kit (DAKO Corporation, Carpinteria, CA, USA).

### 4.19. Statistical Analysis

All data are expressed as mean ± standard deviation (SD). The differences between pairs of groups were analyzed statistically with a student’s *t*-test in Excel (Microsoft, Redmond, WA, USA) or GraphPad Prism 9 software (GraphPad Software Inc., San Diego, CA, USA). *p* < 0.05 was considered statistically significant.

## 5. Conclusions

**Figure 10:**
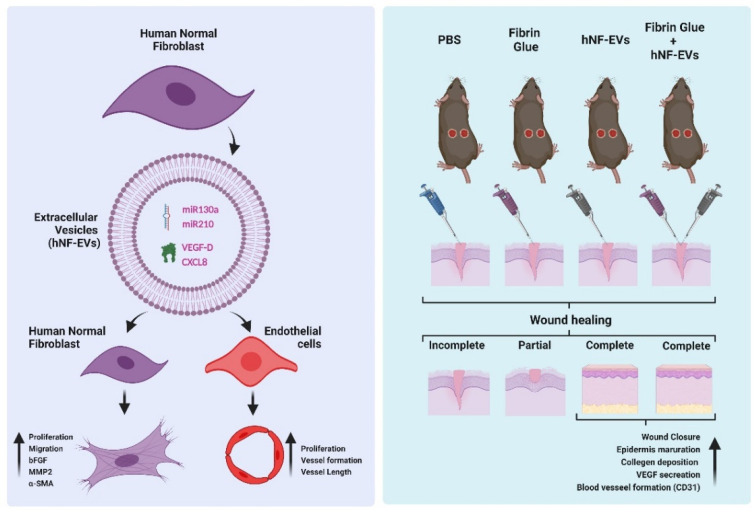
The schematic representation of hNF-EV induced promotion of fibrogenesis and angiogenesis in vitro and accelerated wound healing in in vivo, Created with BioRender.com (accessed on 27 May 2022).

In this study, we isolated and characterized EVs from fibroblasts derived from human dermis. We found that hNF-EVs are enriched with miR130a, miR210, VEGF-D, and CXCL8, which may contribute to the enhanced fibrogenesis and wound healing activities of fibroblasts, in addition to the angiogenic activities of endothelial cells in vitro. The scaffold-free noninvasive delivery of hNF-EVs effectively enhanced the cutaneous wound healing in mice. Finally, hNF-EVs also promoted skin structural development, collagen deposition and maturation, and release of VEGF induced neovascularity (Figure 10). Results of the current study indicate that the scaffold-free noninvasive delivery of hNF-EVs may serve as a new therapeutic tool for cutaneous wound healing.

## Data Availability

Data will be made available upon reasonable request to corresponding authors.

## Supplementary Materials

The following are available online at https://www.mdpi.com/article/10.3390/ph15060702/s1, Figure S1: Confocal microscopy imaging of hNF cells probed with fibroblast marker (vimentin, α-SMA, Hsp47, S100A4) antibodies and secondary Alexa Fluor™ 555 antibody, negative control: no primary antibody was used (scale bar: 20 µm), Figure S2: (A) Representative images display the wound healing process of C57BL/6 mice treated with control: PBS; Fibrin Glue (FG); hNF-EVs; FG + hNF-EVs (day 0, 4, 8, and 16). (B) H&E staining of full wound sections in control: FG, hNF-EV, and FG + hNF-EV groups (day 4, 8, and 16; scale bar = 1 mm).

## Abbreviations

hNF	human normal fibroblast
hNF-EVs	extracellular vesicles from human normal fibroblast
miRNA	microRNA
mRNA	Messenger RNA
TEM	transmission electron microscope
H&E	hematoxylin and eosin
CCK-8	cell counting kit-8
qRT-PCR	quantitative real-time PCR
SDS-PAGE	sodium dodecyl sulfate-polyacrylamide gel electrophoresis

## References

[1] Martin P. (1997). Wound Healing--Aiming for Perfect Skin Regeneration. Science.

[2] Martí-Carvajal A.J., Gluud C., Nicola S., Simancas-Racines D., Reveiz L., Oliva P., Cedeño-Taborda J. (2015). Growth Factors for Treating Diabetic Foot Ulcers. Cochrane Database Syst. Rev..

[3] Choi S.H., Gu J.H., Kang D.H. (2016). Analysis of Traffic Accident-Related Facial Trauma. J. Craniofac. Surg..

[4] Chen L., Xing Q., Zhai Q., Tahtinen M., Zhou F., Chen L., Xu Y., Qi S., Zhao F. (2017). Pre-Vascularization Enhances Therapeutic Effects of Human Mesenchymal Stem Cell Sheets in Full Thickness Skin Wound Repair. Theranostics.

[5] Ryu J.Y., Lee J.H., Kim J.S., Lee J.S., Lee J.W., Choi K.Y., Chung H.Y., Cho B.C., Yang J.D. (2021). Usefulness of Incisional Negative Pressure Wound Therapy for Decreasing Wound Complication Rates and Seroma Formation Following Prepectoral Breast Reconstruction. Aesthetic Plast. Surg..

[6] Oh E.J., Gangadaran P., Rajendran R.L., Kim H.M., Oh J.M., Choi K.Y., Chung H.Y., Ahn B.-C. (2021). Extracellular Vesicles Derived from Fibroblasts Promote Wound Healing by Optimizing Fibroblast and Endothelial Cellular Functions. Stem Cells.

[7] Gonzalez A.C., Costa T.F., Andrade Z.D., Medrado A.R. (2016). Wound Healing—A Literature Review. Bras. Derm..

[8] Eming S.A., Martin P., Tomic-Canic M. (2014). Wound Repair and Regeneration: Mechanisms, Signaling, and Translation. Sci. Transl. Med..

[9] Guo S., DiPietro L.A. (2010). Factors Affecting Wound Healing. J. Dent. Res..

[10] Chittoria R.K., Kumar S.H., Shiffman M.A., Low M. (2021). Low-Level Laser Therapy (LLLT) in Wound Healing. Chronic Wounds, Wound Dressings and Wound Healing.

[11] Hopkins J.T., McLoda T.A., Seegmiller J.G., David Baxter G. (2004). Low-Level Laser Therapy Facilitates Superficial Wound Healing in Humans: A Triple-Blind, Sham-Controlled Study. J. Athl. Train.

[12] Goldman R. (2004). Growth Factors and Chronic Wound Healing: Past, Present, and Future. Adv. Ski. Wound Care.

[13] Herskovitz I., Hughes O.B., Macquhae F., Rakosi A., Kirsner R. (2016). Epidermal Skin Grafting. Int. Wound J..

[14] Oh E.J., Lee H.W., Kalimuthu S., Kim T.J., Kim H.M., Baek S.H., Zhu L., Oh J.M., Son S.H., Chung H.Y. (2018). In Vivo Migration of Mesenchymal Stem Cells to Burn Injury Sites and Their Therapeutic Effects in a Living Mouse Model. J. Control. Release.

[15] Kosaric N., Kiwanuka H., Gurtner G.C. (2019). Stem Cell Therapies for Wound Healing. Expert Opin. Biol..

[16] Nourian Dehkordi A., Mirahmadi Babaheydari F., Chehelgerdi M., Raeisi Dehkordi S. (2019). Skin Tissue Engineering: Wound Healing Based on Stem-Cell-Based Therapeutic Strategies. Stem Cell Res. Ther..

[17] Nawaz M., Fatima F., Vallabhaneni K.C., Penfornis P., Valadi H., Ekström K., Kholia S., Whitt J.D., Fernandes J.D., Pochampally R. (2015). Extracellular Vesicles: Evolving Factors in Stem Cell Biology. Stem Cells Int..

[18] Gangadaran P., Rajendran R.L., Oh J.M., Hong C.M., Jeong S.Y., Lee S.-W., Lee J., Ahn B.-C. (2020). Extracellular Vesicles Derived from Macrophage Promote Angiogenesis In Vitro and Accelerate New Vasculature Formation In Vivo. Exp. Cell Res..

[19] Kwack M.H., Seo C.H., Gangadaran P., Ahn B.-C., Kim M.K., Kim J.C., Sung Y.K. (2019). Exosomes Derived from Human Dermal Papilla Cells Promote Hair Growth in Cultured Human Hair Follicles and Augment the Hair-Inductive Capacity of Cultured Dermal Papilla Spheres. Exp. Derm..

[20] Gangadaran P., Rajendran R.L., Lee H.W., Kalimuthu S., Hong C.M., Jeong S.Y., Lee S.-W., Lee J., Ahn B.-C. (2017). Extracellular Vesicles from Mesenchymal Stem Cells Activates VEGF Receptors and Accelerates Recovery of Hindlimb Ischemia. J. Control. Release.

[21] Gangadaran P., Ahn B.-C. (2020). Extracellular Vesicle- and Extracellular Vesicle Mimetics-Based Drug Delivery Systems: New Perspectives, Challenges, and Clinical Developments. Pharmaceutics.

[22] Gangadaran P., Hong C.M., Ahn B.-C. (2018). An Update on in Vivo Imaging of Extracellular Vesicles as Drug Delivery Vehicles. Front. Pharm..

[23] Fuloria S., Subramaniyan V., Dahiya R., Dahiya S., Sudhakar K., Kumari U., Sathasivam K., Meenakshi D.U., Wu Y.S., Sekar M. (2021). Mesenchymal Stem Cell-Derived Extracellular Vesicles: Regenerative Potential and Challenges. Biology.

[24] Rani S., Ryan A.E., Griffin M.D., Ritter T. (2015). Mesenchymal Stem Cell-Derived Extracellular Vesicles: Toward Cell-Free Therapeutic Applications. Mol. Ther..

[25] Tsiapalis D., O’Driscoll L. (2020). Mesenchymal Stem Cell Derived Extracellular Vesicles for Tissue Engineering and Regenerative Medicine Applications. Cells.

[26] Hu Y., Rao S.-S., Wang Z.-X., Cao J., Tan Y.-J., Luo J., Li H.-M., Zhang W.-S., Chen C.-Y., Xie H. (2018). Exosomes from Human Umbilical Cord Blood Accelerate Cutaneous Wound Healing through MiR-21-3p-Mediated Promotion of Angiogenesis and Fibroblast Function. Theranostics.

[27] Zhang J., Guan J., Niu X., Hu G., Guo S., Li Q., Xie Z., Zhang C., Wang Y. (2015). Exosomes Released from Human Induced Pluripotent Stem Cells-Derived MSCs Facilitate Cutaneous Wound Healing by Promoting Collagen Synthesis and Angiogenesis. J. Transl. Med..

[28] Zhang B., Wang M., Gong A., Zhang X., Wu X., Zhu Y., Shi H., Wu L., Zhu W., Qian H. (2015). HucMSC-Exosome Mediated-Wnt4 Signaling Is Required for Cutaneous Wound Healing. Stem Cells.

[29] Liu X., Yang Y., Li Y., Niu X., Zhao B., Wang Y., Bao C., Xie Z., Lin Q., Zhu L. (2017). Integration of Stem Cell-Derived Exosomes with in Situ Hydrogel Glue as a Promising Tissue Patch for Articular Cartilage Regeneration. Nanoscale.

[30] Shabbir A., Cox A., Rodriguez-Menocal L., Salgado M., Badiavas E.V. (2015). Mesenchymal Stem Cell Exosomes Induce Proliferation and Migration of Normal and Chronic Wound Fibroblasts, and Enhance Angiogenesis In Vitro. Stem Cells Dev..

[31] de Mayo T., Conget P., Becerra-Bayona S., Sossa C.L., Galvis V., Arango-Rodríguez M.L. (2017). The Role of Bone Marrow Mesenchymal Stromal Cell Derivatives in Skin Wound Healing in Diabetic Mice. PLoS ONE.

[32] Li X., Liu L., Yang J., Yu Y., Chai J., Wang L., Ma L., Yin H. (2016). Exosome Derived From Human Umbilical Cord Mesenchymal Stem Cell Mediates MiR-181c Attenuating Burn-Induced Excessive Inflammation. EBioMedicine.

[33] Rajendran R.L., Gangadaran P., Bak S.S., Oh J.M., Kalimuthu S., Lee H.W., Baek S.H., Zhu L., Sung Y.K., Jeong S.Y. (2017). Extracellular Vesicles Derived from MSCs Activates Dermal Papilla Cell in Vitro and Promotes Hair Follicle Conversion from Telogen to Anagen in Mice. Sci Rep..

[34] Saraswati S., Marrow S.M.W., Watch L.A., Young P.P. (2019). Identification of a Pro-Angiogenic Functional Role for FSP1-Positive Fibroblast Subtype in Wound Healing. Nat. Commun..

[35] Xu J., Liu X., Koyama Y., Wang P., Lan T., Kim I.-G., Kim I.H., Ma H.-Y., Kisseleva T. (2014). The Types of Hepatic Myofibroblasts Contributing to Liver Fibrosis of Different Etiologies. Front. Pharm..

[36] Gangadaran P., Rajendran R.L., Oh J.M., Oh E.J., Hong C.M., Chung H.Y., Lee J., Ahn B.-C. (2021). Identification of Angiogenic Cargo in Extracellular Vesicles Secreted from Human Adipose Tissue-Derived Stem Cells and Induction of Angiogenesis In Vitro and In Vivo. Pharmaceutics.

[37] Munir J., Yoon J.K., Ryu S. (2020). Therapeutic MiRNA-Enriched Extracellular Vesicles: Current Approaches and Future Prospects. Cells.

[38] DiPietro L.A. (2016). Angiogenesis and Wound Repair: When Enough Is Enough. J. Leukoc. Biol..

[39] Chen Y., Gorski D.H. (2008). Regulation of Angiogenesis through a MicroRNA (MiR-130a) That down-Regulates Antiangiogenic Homeobox Genes GAX and HOXA5. Blood.

[40] Yang H., Zhang H., Ge S., Ning T., Bai M., Li J., Li S., Sun W., Deng T., Zhang L. (2018). Exosome-Derived MiR-130a Activates Angiogenesis in Gastric Cancer by Targeting C-MYB in Vascular Endothelial Cells. Mol. Ther..

[41] Liu F., Lou Y.-L., Wu J., Ruan Q.-F., Xie A., Guo F., Cui S.-P., Deng Z.-F., Wang Y. (2012). Upregulation of MicroRNA-210 Regulates Renal Angiogenesis Mediated by Activation of VEGF Signaling Pathway under Ischemia/Perfusion Injury in Vivo and in Vitro. KBR.

[42] Fan Z.-G., Qu X.-L., Chu P., Gao Y.-L., Gao X.-F., Chen S.-L., Tian N.-L. (2018). MicroRNA-210 Promotes Angiogenesis in Acute Myocardial Infarction. Mol. Med. Rep..

[43] Narayanan S., Eliasson Angelstig S., Xu C., Grünler J., Zhao A., Zhu W., Xu Landén N., Ståhle M., Zhang J., Ivan M. (2020). HypoxamiR-210 Accelerates Wound Healing in Diabetic Mice by Improving Cellular Metabolism. Commun. Biol..

[44] Vallabhaneni K.C., Penfornis P., Dhule S., Guillonneau F., Adams K.V., Mo Y.Y., Xu R., Liu Y., Watabe K., Vemuri M.C. (2014). Extracellular Vesicles from Bone Marrow Mesenchymal Stem/Stromal Cells Transport Tumor Regulatory MicroRNA, Proteins, and Metabolites. Oncotarget.

[45] Shimoda M., Khokha R. (2017). Metalloproteinases in Extracellular Vesicles. Biochim. Biophys. Acta (BBA)-Mol. Cell Res..

[46] Rissanen T.T., Markkanen J.E., Gruchala M., Heikura T., Puranen A., Kettunen M.I., Kholová I., Kauppinen R.A., Achen M.G., Stacker S.A. (2003). VEGF-D Is the Strongest Angiogenic and Lymphangiogenic Effector Among VEGFs Delivered Into Skeletal Muscle via Adenoviruses. Circ. Res..

[47] Bower N.I., Vogrin A.J., Le Guen L., Chen H., Stacker S.A., Achen M.G., Hogan B.M. (2017). Vegfd Modulates Both Angiogenesis and Lymphangiogenesis during Zebrafish Embryonic Development. Development.

[48] Li A., Dubey S., Varney M.L., Dave B.J., Singh R.K. (2003). IL-8 Directly Enhanced Endothelial Cell Survival, Proliferation, and Matrix Metalloproteinases Production and Regulated Angiogenesis. J. Immunol..

[49] Rennekampff H.-O., Hansbrough J.F., Kiessig V., Doré C., Sticherling M., Schröder J.-M. (2000). Bioactive Interleukin-8 Is Expressed in Wounds and Enhances Wound Healing. J. Surg. Res..

[50] Nakamichi M., Akishima-Fukasawa Y., Fujisawa C., Mikami T., Onishi K., Akasaka Y. (2016). Basic Fibroblast Growth Factor Induces Angiogenic Properties of Fibrocytes to Stimulate Vascular Formation during Wound Healing. Am. J. Pathol..

[51] Li B., Wang J.H.-C. (2011). Fibroblasts and Myofibroblasts in Wound Healing: Force Generation and Measurement. J. Tissue Viability.

[52] Nguyen T.T., Mobashery S., Chang M. (2016). Roles of Matrix Metalloproteinases in Cutaneous Wound Healing.

[53] Rundhaug J.E. (2005). Matrix Metalloproteinases and Angiogenesis. J. Cell. Mol. Med..

[54] Shinde A.V., Humeres C., Frangogiannis N.G. (2017). The Role of α-Smooth Muscle Actin in Fibroblast-Mediated Matrix Contraction and Remodeling. Biochim. Biophys. Acta (BBA) Mol. Basis Dis..

[55] Hinz B., Celetta G., Tomasek J.J., Gabbiani G., Chaponnier C. (2001). Alpha-Smooth Muscle Actin Expression Upregulates Fibroblast Contractile Activity. MBoC.

[56] Wang S., Li X., Parra M., Verdin E., Bassel-Duby R., Olson E.N. (2008). Control of Endothelial Cell Proliferation and Migration by VEGF Signaling to Histone Deacetylase 7. Proc. Natl. Acad. Sci. USA.

[57] Casado-Díaz A., Quesada-Gómez J.M., Dorado G. (2020). Extracellular Vesicles Derived From Mesenchymal Stem Cells (MSC) in Regenerative Medicine: Applications in Skin Wound Healing. Front. Bioeng. Biotechnol..

[58] Chen Y., Yu Q., Xu C.-B. (2017). A Convenient Method for Quantifying Collagen Fibers in Atherosclerotic Lesions by ImageJ Software. Int. J. Clin. Exp. Med..

[59] Wu J., Zhu J., He C., Xiao Z., Ye J., Li Y., Chen A., Zhang H., Li X., Lin L. (2016). Comparative Study of Heparin-Poloxamer Hydrogel Modified BFGF and AFGF for in Vivo Wound Healing Efficiency. ACS Appl Mater. Interfaces.

[60] Singer A.J., Clark R.A. (1999). Cutaneous Wound Healing. N. Engl. J. Med..

[61] Li Y., Zhang J., Shi J., Liu K., Wang X., Jia Y., He T., Shen K., Wang Y., Liu J. (2021). Exosomes Derived from Human Adipose Mesenchymal Stem Cells Attenuate Hypertrophic Scar Fibrosis by MiR-192-5p/IL-17RA/Smad Axis. Stem Cell Res..

[62] Wang L., Hu L., Zhou X., Xiong Z., Zhang C., Shehada H.M.A., Hu B., Song J., Chen L. (2017). Exosomes Secreted by Human Adipose Mesenchymal Stem Cells Promote Scarless Cutaneous Repair by Regulating Extracellular Matrix Remodelling. Sci. Rep..

[63] Li C., Wei S., Xu Q., Sun Y., Ning X., Wang Z. (2021). Application of ADSCs and Their Exosomes in Scar Prevention. Stem Cell Rev. Rep..

[64] Yoon D., Yoon D., Sim H., Hwang I., Lee J.-S., Chun W. (2018). Accelerated Wound Healing by Fibroblasts Differentiated from Human Embryonic Stem Cell-Derived Mesenchymal Stem Cells in a Pressure Ulcer Animal Model. Stem Cells Int..

[65] Gangadaran P., Li X.J., Lee H.W., Oh J.M., Kalimuthu S., Rajendran R.L., Son S.H., Baek S.H., Singh T.D., Zhu L. (2017). A New Bioluminescent Reporter System to Study the Biodistribution of Systematically Injected Tumor-Derived Bioluminescent Extracellular Vesicles in Mice. Oncotarget.

[66] Théry C., Amigorena S., Raposo G., Clayton A. (2006). Isolation and Characterization of Exosomes from Cell Culture Supernatants and Biological Fluids. Curr. Protoc. Cell Biol..

[67] Zudaire E., Gambardella L., Kurcz C., Vermeren S. (2011). A Computational Tool for Quantitative Analysis of Vascular Networks. PLoS ONE.

[68] Park G.Y., Yeum J.H., Yang D.J., Park G.O., Kim Y.H., Jeon S., Kim T.J., Oh E.J., Chung H.Y., Choi J.H. (2018). Moisture Wound Healing Characteristics of Alginate Sponge and Hydrogel. Polymer.

[69] Ranganathan K., Kavitha R., Sawant S.S., Vaidya M.M. (2006). Cytokeratin Expression in Oral Submucous Fibrosis—An Immunohistochemical Study. J. Oral Pathol. Med..

