# Supplementary material for: Identification of Angiogenic Cargoes in Human Fibroblasts-Derived Extracellular Vesicles and Induction of Wound Healing
Source: Pharmaceuticals (Basel). 2022 Jun 2;15(6):702. doi: 10.3390/ph15060702 (PMC9230817; doi:10.3390/ph15060702)
Supplement: Supplementary file 1 [file pharmaceuticals-15-00702-s001.zip › pharmaceuticals-1726026-supplementary.pdf]

Identification of Angiogenic Cargoes in Human Fibroblasts-Derived Extracellular Vesicles and Induction of Wound Healing

Prakash Gangadaran [1,2,†], Eun Jung Oh [3,†], Ramya Lakshmi Rajendran [2], Hyun Mi Kim [3], Ji Min Oh [2], Suin Kwak [1,3], Chae Moon Hong [2,4], Kang Young Choi [3], Ho Yun Chung [1,3,*] and Byeong-Cheol Ahn [1,2,4,*]

[1] BK21 FOUR KNU Convergence Educational Program of Biomedical Sciences for Creative Future Talents,
Department of Biomedical Science, School of Medicine, Kyungpook National University, Daegu 41944, Korea;
prakashg@knu.ac.kr (P.G.); suin8349@naver.com (K.S.)

[2] Department of Nuclear Medicine, School of Medicine, Kyungpook National University, Daegu 41944, Korea;
ramyag@knu.ac.kr (R.L.R.); ojm0366@knu.ac.kr (J.M.O.); cmhong@knu.ac.kr (C.M.H.)

[3] Department of Plastic and Reconstructive Surgery, CMRI, School of Medicine, Kyungpook National
University, Kyungpook National University Hospital, Daegu 41944, Korea;
fullrest74@knu.ac.kr (E.J.O.);
sarang7939@naver.com (H.M.K.); kychoi@knu.ac.kr (K.Y.C.)

[4] Department of Nuclear Medicine, School of Medicine, Kyungpook National University Hospital ,
Daegu 41944, Korea

* Correspondence: hy-chung@knu.ac.kr (H.Y.C.); abc2000@knu.ac.kr (B.-C.A.); Tel.: +82-53-420-5692 (H.Y.C.);
+82-53-420-5583 (B.-C.A.); Fax: +82-53-425-3879 (H.Y.C.); +82-53-200-6447 (B.-C.A.)

† These authors contributed equally to this work.

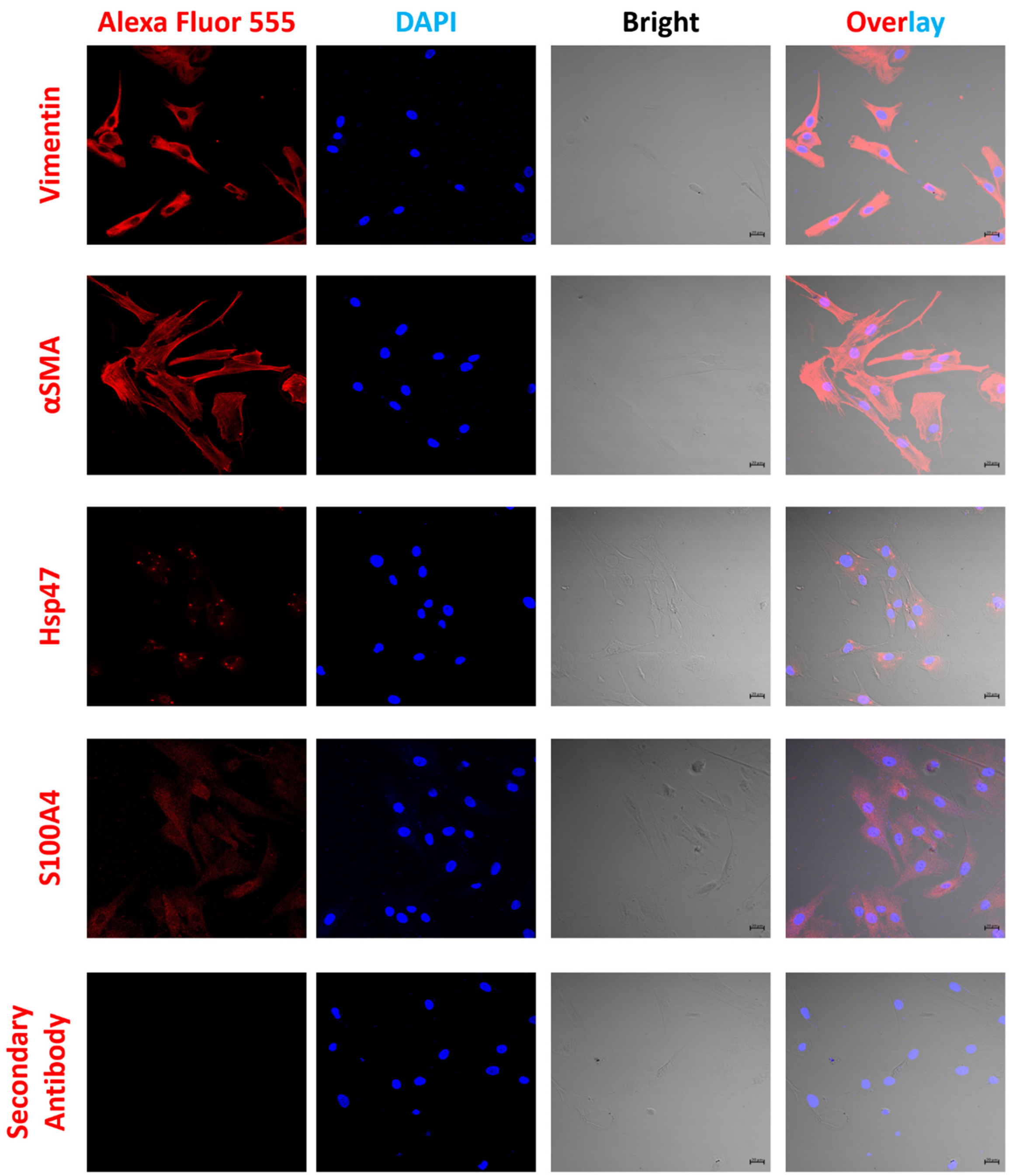

Supplementary Figure S1: Confocal microscopy imaging of hNF cells probed with fibroblast markers (Vimentin, α-SMA, Hsp47, S100A4) antibodies and secondary Alexa Fluor™ 555 antibody, negative control: no primary antibody was used (scale bar: 20 µm).

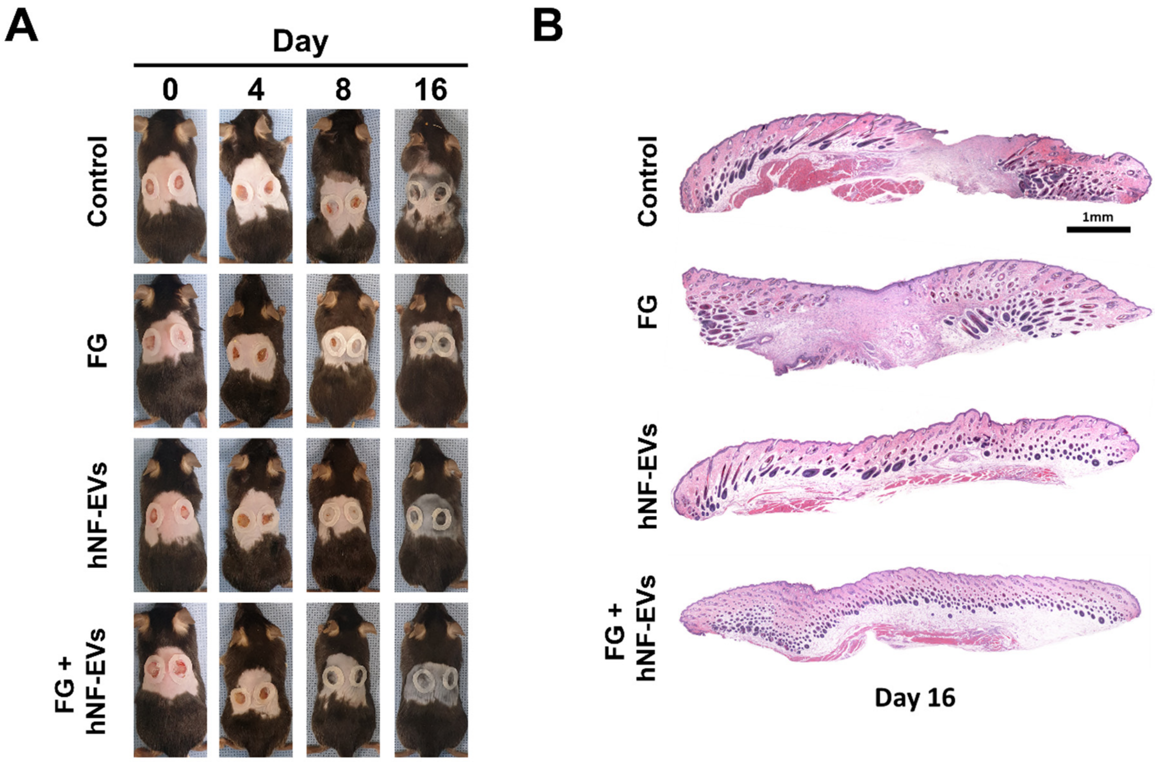

Supplementary Figure S2: (A) Representative images display the wound healing process of C57BL/6 mice treated with Control: PBS; Fibrin Glue: FG; hNF-EVs; FG + hNF-EVs (day 0, 4, 8, and 16). **(B)** H&E staining of full wound sections in Control: FG; hNF-EVs and FG + hNF-EVs groups (day 4, 8, and 16; scale bar = 1 mm).
